# Assessment of an undergraduate psychiatry course in an African setting

**DOI:** 10.1186/1472-6920-8-23

**Published:** 2008-04-22

**Authors:** Benjamin J Baig, Anna Beaglehole, Robert C Stewart, Leonie Boeing, Douglas H Blackwood, Johan Leuvennink, Felix Kauye

**Affiliations:** 1 Scotland-Malawi Mental Health Education Project, Kennedy Tower, Royal Edinburgh Hospital, Morningside Park, Edinburgh, EH10 5HF, UK; 2 Chief Government Psychiatrist (Ministry of Health, Malawi) and Visiting Clinical Lecturer, College of Medicine, University of Malawi, Private Bag 360, Chichiri, Blantyre 3, Malawi

## Abstract

**Background:**

International reports recommend the improvement in the amount and quality of training for mental health workers in low and middle income countries. The Scotland-Malawi Mental Health Education Project (SMMHEP) has been established to support the teaching of psychiatry to medical students in the University of Malawi. While anecdotally supportive medical educational initiatives appear of value, little quantitative evidence exists to demonstrate whether such initiatives can deliver comparable educational standards. This study aimed to assess the effectiveness of an undergraduate psychiatry course given by UK psychiatrists in Malawi by studying University of Malawi and Edinburgh University medical students' performance on an MCQ examination paper.

**Methods:**

An undergraduate psychiatry course followed by an MCQ exam was delivered by the SMMHEP to 57 Malawi medical students. This same MCQ exam was given to 71 Edinburgh University medical students who subsequently sat their own Edinburgh University examination.

**Results:**

There were no significant differences between Edinburgh students' performance on the Malawi exam and their own Edinburgh University exam. (p = 0.65). This would suggest that the Malawi exam is a comparable standard to the Edinburgh exam. Malawi students marks ranged from 52.4%–84.6%. Importantly 84.4% of Malawi students scored above 60% on their exam which would equate to a hypothetical pass by UK university standards.

**Conclusion:**

The support of an undergraduate course in an African setting by high income country specialists can attain a high percentage pass rate by UK standards. Although didactic teaching has been surpassed by more novel educational methods, in resource poor countries it remains an effective and cost effective method of gaining an important educational standard.

## Background

In low and middle income countries, mental health is less recognized and mental health staff are often inadequately trained to deal with the growing problem. [1] Concern about the global burden of mental health has prompted three high profile international reports. [2-4] In these reports many recommendations were made including the improvement in the amount and quality of training for all mental health workers, the development of local human resources and the need to link with institutions in high income countries.

A further issue in the delivery of healthcare in low and middle income countries relates to the "brain-drain" of health care professionals. [5,6] The establishment of medical schools in such countries has had a positive effect in encouraging locally trained physicians to work in their home country. [7] The advantages of local training of medical professionals, and the difficulties posed by scarce resources, means that there is a need for high income countries to support education delivery in low and middle income countries.

Medical education in Africa is evolving from didactic teaching to incorporate community-based education, problem-based learning, and integrated teaching. [8,9] This process has been facilitated through external aid funding from resource rich countries. However, when resources are limited, lecture-based teaching provides a financially easier solution. [10]

The medical school in Malawi was set up in 1991 by the University of Malawi with support from the British, Dutch and German governments and the World Health Organization. In the first 10 years, 168 doctors graduated from the medical school. [7] Many initiatives have supported medical education at the College of Medicine, University of Malawi in the past 15 years but an ongoing concern has related to teaching in mental health. [7]

The Scotland-Malawi Mental Health Education Project (SMMHEP) was established in 2005 to support the teaching of psychiatry to medical students in the College of Medicine. The project has been working in conjunction with the College of Medicine, and works in close collaboration with local clinicians and institutions. It supports delivery of a theoretical knowledge base in psychiatry to establish basic clinical skills and to stimulate discussion about mental illness amongst the undergraduate students. [11]

Before 2005 the undergraduate psychiatry module was delivered at times by a single lecturer and provided variable clinical experience to the students. There have been several previous attempts to design a sustainable psychiatry teaching program in Malawi. [12] At present there is only one trained psychiatrist in the country, so a model in which he was supported by external trainers visiting Malawi for the period of teaching module provided a possible solution. [11]

An inherent difficulty in delivering an undergraduate psychiatry course in this way is the issue of transcultural specificity of illness presentation and management. Psychiatric illness will be more subject to cultural difference and hence course design must accommodate this. Some authors note that using western curricula in this instance is inappropriate. [12] However, core psychiatry including pharmacology, genetics, neuropsychiatry, classification of illness and biological treatment could be seen as generalisable. [13]

In spite of the many initiatives that have supported medical education in Africa, it has often been difficult to measure the standard of education delivered. While many of the African medical schools (including the College of Medicine) are WHO accredited, it is difficult to assess how the standard of individual undergraduate courses compares with their equivalents in high income countries. Importantly, SMMEHP wanted to assess whether the course design would lead to successful examination performance.

This paper assesses the value of a 2-week undergraduate theoretical psychiatry course given through SMMHEP and measures how the teaching standard compares with UK university standard. It was hypothesized that the course could bring students to a level of knowledge approaching the standard that courses would attain in the UK.

## Methods

There were two components to this study: firstly the delivery of theoretical teaching and an examination to undergraduates in Malawi, and secondly the delivery of the same examination to undergraduates in Edinburgh, UK. The grades for the Multiple Choice Questions from the theoretical exam were chosen to be the primary outcome measure.

### Malawi

At the time of the study, the psychiatry curriculum at the College of Medicine, University of Malawi consisted of two weeks theoretical teaching in the third year followed by a five week psychiatry placement and assessment in the fourth year. The third year program included a basic introduction to psychiatry. The fourth year course consisted of two weeks of lectures and tutorials following which the students receive a theoretical exam. The theoretical teaching was followed by a three week clinical attachment in which students acquired clinical skills. At the end of the clinical attachment was a final clinical oral exam. The assessment of the students was made up of grades from the theoretical exam, clinical attachment grade (which included attendance, participation and clinical skills), clinical oral exam and a written clinical case. The exam which the students sat after their two week period of theoretical teaching consisted of Multiple Choice Questions and three short essay questions.

The theoretical and clinical teaching was delivered by the Clinical Lecturer, College of Medicine and volunteer teaching staff from SMMHEP. The SMHEPP teaching staff consisted of 1 Senior Lecturer (University of Edinburgh), 1 Consultant and 4 Specialist Registrars. All teaching staff were involved in delivering undergraduate education to fourth year medical students in Scottish medical schools. The teaching was also supported by a volunteer consultant psychiatrist from Norway.

In the 2007 year group there were 57 4th year medical students (15 female, 42 Male). All students had successfully passed their first three years of medical school and were accustomed to the teaching and examination format they received in psychiatry.

It is important to note that the Malawi students had only two weeks of teaching prior to this theoretical exam and three days to study for it, following the teaching.

### Edinburgh

The psychiatry course in the University of Edinburgh medical school at the time of the study consists of clinical and theoretical teaching in the undergraduate third and fourth years. The third year course, which is spread over 5 months, consisted of theoretical lecture teaching and introductory tutorials focusing on clinical interviewing. The fourth year experience in psychiatry consisted of a seven week clinical attachment, usually in two clinical settings during which the students receive ward based teaching along with standardized seminars and tutorials. Here the teaching is both didactic and clinical. Following the clinical attachment the students were given a theoretical exam consisting of Multiple Choice Questions followed by a clinical oral exam.

### Examination

The theoretical examination undertaken by the Malawi students was written by the teaching staff from SMMHEP. The questions were based on an undergraduate psychiatry standard from the UK. The questions reflected the taught syllabus and emphasized the cultural context of clinical psychiatry in Malawi. The exam was made up of 40 5-stem True/False questions and 16 Single Best Answer questions. It was given after the two week theoretical teaching in the 4th year course. These were delivered under exam conditions and candidates had 90 minutes to complete this part of the exam.

This same exam paper was given to the fourth year Edinburgh University students. It was delivered on the last day of their 7 week psychiatry attachment and three days prior to their Edinburgh University MCQ exam.

The Malawi exam was sat in Edinburgh under exam conditions and participants had 90 minutes to complete the paper. The exam was marked by the university standardized marking system.

### Ethical approval

Ethical approval in Edinburgh was gained from the University of Edinburgh College Committee on the Use of Student Volunteers for this project. Ethical approval in Malawi was gained from the College of Medicine Research and Ethics Committee (COMREC).

## Results

The Malawi exam was sat by 57 students from the College of Medicine, University of Malawi and 71 students from the University of Edinburgh. The Malawi students were similar to Edinburgh students with regards to age and previous experience of examinations in medical school (Table 1).

The Malawi students sat their exam three days after their two week theoretical course; their marks ranged from 52.4 to 84.6% in their MCQ exam with the average of 72.7%. Of 81 Edinburgh students in their psychiatry group, 71 (87.7%) agreed to participate in the Malawi exam. These Edinburgh students sat the exam on the last day of their 7 week clinical attachment and three days before their actual Edinburgh MCQ exam. As can be seen from table 2, marks ranged from 67.5–89.4%.

**Table 1:** The table shows demographic and educational indices of Edinburgh and Malawi medical students

	Malawi Students	Edinburgh Students
Gender (% male)	67.6%	36.3%
Age (mean and range)	21.7 (21–24)	22.2 (21–27)
University Entrance Requirements	At least six O-level passes including English and Mathematics; and three A-level passes with a grade of at least C in Biology, Chemistry and one other science subject	3 A-levels and 1 AS in 4 subjects Chemistry at A-level Maths or Physics or Biology at A-level Biology at least at AS level Grades AAAB
English as first language (%)	15% (All students were required to be fluent in English as a course requirement)	94%
MCQ exams in medical school	MCQ format used in most clinical and preclinical exams	MCQ format used in most clinical and preclinical exams

**Table 2:** The table shows descriptive statistics of Edinburgh and Malawi students' performance in the MCQ exams

**Group**	**Number**	**M C Q Average mark**	**Standard deviation**	**Range**	**Inter group Comparison**
1. Malawi Students sitting Malawi Exam	57	72.7%	3.51	52.4–84.6%	
2. Edinburgh Students sitting Malawi Exam	71	82.5%	5.65	67.5–89.4%	Group 1 vs Group 2 **p = 0.04 **(Independent T-test)
3. Edinburgh Students sitting Edinburgh Exam	71	86.6%	5.37	78%–96.4%	Group 2 vs Group 3 **p = 0.65 **(Paired T-test)

When the Edinburgh students sat their own MCQ the marks ranged from 78%–96.4% with an average of 86%. Importantly there is no significant difference between the marks from the Malawi exam and the Edinburgh exam sat by Edinburgh students. It can be argued that the Malawi MCQ exam is not significantly different in standard to the Edinburgh MCQ exam. If this is assumed to be the case, the Malawi exam could be hypothetically extrapolated to be marked by an Edinburgh University marking system in which 90–100% would be grade "A", 80–90% a grade "B" etc. The pass mark is 60% or grade D.

If we then measured the success of the teaching course in Malawi by the extrapolated Edinburgh University grades we would find that of 57 Malawi students; 3 would get grade B, 30 would get grade C, 15 would get D and 9 grade E (by Edinburgh University marking system). By these standards, after the brief intensive psychiatry course provided, 48 (84.4%) of the Malawi students could have deemed to pass the exam according to a hypothetical UK university marking standard.

## Discussion

The main aims of SMMHEP are to support the sole Malawian psychiatrist in delivering an undergraduate course and examination system in psychiatry to medical students in Malawi. The primary aim of this study was to demonstrate that the standard of exam in Malawi was comparable to that given to UK students in Edinburgh. Given that, amongst the Edinburgh students, there was no significant difference in marks achieved in the formal Edinburgh University and the Malawi exam, it can be argued that this is the case.

The secondary aim of this evaluation was to assess the success of a 2 week lecture based theoretical psychiatry course in this setting. Given the very different nature of the Malawian and Edinburgh teaching modules, no comparison can (or should) be made between the academic standards of the individual students themselves. However, it can be argued that an MCQ exam is a good measure of the knowledge acquisition associated with a teaching intervention, and that those Malawi students who reached a reasonable grade in this MCQ examination had obtained and demonstrated proficiency in theoretical understanding of psychiatry. It is acknowledged that Edinburgh and Malawi students are demographically different and will have been exposed to significantly different secondary and tertiary educational systems. Given that Edinburgh students have amongst the highest entrance requirements in the UK and had several months of didactic and clinical teaching compared to the two weeks that the Malawi students received, we feel that an extremely valuable standard was reached by Malawi students. We conclude that a 2 week lecture-based course is a valid educational intervention for delivering a standard of knowledge comparable to UK university standards.

This finding is important for several reasons. As has been discussed, in low and middle income countries the opportunity to deliver computer assisted, problem based learning and integrated teaching is extremely limited. While didactic teaching has been superseded by more advanced educational methods, it can be argued that, where resources are limited, lecture based teaching still has a place in obtaining a good undergraduate standard. As Malawi is unlikely to be able to significantly increase its resources for undergraduate psychiatry teaching, evidence must be found to validate educational methods which are instigated by high income countries. The use of 5 clinician/academics from the UK to support a two week project leading to a hypothetical 84.4% exam pass rate can be seen as an example of justifiable use of resources for a measurable conclusion. Data such as this is essential to justify initiatives from high income countries.

The use of a multiple choice format as a comparator of standards poses some limitations. The exposure to MCQ formats may be different between Edinburgh and Malawi but both groups had previously done medical examinations in this format. The above findings only suggest that theoretical knowledge can be compared between Malawi and UK students. This study does not suggest that teaching of clinical knowledge and clinical ability are of equivalent standard. Both Edinburgh and Malawi students received a clinical viva exam in addition to their written paper. While all Malawi students were deemed to have passed their clinical viva, the examination process for this format depends on subjective assessment by examiners and would not be deemed a valid comparator.

A further limitation to this methodology relates to the course content and examination content. In order to avoid implementing a westernized model of psychiatry, some of the Malawi course related to psychiatric issues which would predominate in an African setting. For example, these would include the relationship between mental illness and HIV. While this topic featured more heavily in the Malawi MCQ exam, it also features on the curriculum in the UK. Thus, although curriculum emphasis may have differed, core content was similar. One specific modification between the exam sat by Malawi students and Edinburgh students related to methods of suicide. Here, students were asked which the most common method in their respective country was. While this was a difference between the exams it measured the same knowledge yet maintained awareness of cultural specificity.

A criticism of the methodology in this study may be that Edinburgh students knowingly sat the Malawi exam as a mock exam and consequently made less effort in their performance. Short of giving Malawi students the exact Edinburgh exam paper, which would not have been possible (due to Edinburgh University restrictions), this limitation was unavoidable. It can be noted however that Edinburgh students sat this exam within several days of their own paper which should mean that they were performing at a similar standard to that they performed in their own exam. Furthermore, Edinburgh students were allowed the same timeframe to sit the Malawi paper and few finished or left the exam hall before the allotted time. This could suggest that the similar effort was placed in performance.

## Conclusion

While there exists limitations in this study, multiple choice question format remains an objective assessment of teaching standards. Success of international development projects is difficult to measure, yet it is essential that some measurement underpins the justification for funding such projects. To date, no studies have been published which directly study standards of low income and high income countries in terms of medical undergraduate education. It remains essential for medical education in low and middle income countries for evidence based initiatives to support local teaching and supplement resources. As such, we believe that this initiative can demonstrate a measurable educational outcome and its format could be generalized to other settings.

## Abbreviations

SMMHEP: Scotland Malawi Mental Health Education Project; MCQ: Multiple Choice Questions.

## Competing interests

The author(s) declares that they have no competing interests.

## Authors' contributions

BB designed the study, wrote and carried out the Edinburgh MCQ exam and drafted the manuscript. AB carried out the Edinburgh MCQ exam and made significant contributions to writing the study. RS, LB, JL and DB made significant contributions to marking the Malawi MCQ and writing the manuscript. FK coordinated and lead the Malawi MCQ exam and made significant contributions to writing the study. All authors read and approved the final manuscript.

## Pre-publication history

The pre-publication history for this paper can be accessed here:


